# Development and validation of the rest experience scale for adults with chronically fatiguing conditions

**DOI:** 10.1371/journal.pone.0355730

**Published:** 2026-09-02

**Authors:** Martin Ackah, Ulric S. Abonie, Florentina Johanna Hettinga, Katie L. Hackett, Vincent Deary

**Affiliations:** 1 Department of Sport Exercise and Rehabilitation, Northumbria University, Newcastle upon Tyne, United Kingdom; 2 Department of Human Movement Sciences, Vrije Universiteit Amsterdam, Amsterdam, The Netherlands; 3 Department of Social Work, Education and Community Wellbeing, Northumbria University, Newcastle upon Tyne, United Kingdom; 4 Department of Psychology, Northumbria University, Newcastle upon Tyne, United Kingdom; Charite Universitatsmedizin Berlin, GERMANY

## Abstract

**Introduction:**

Rest is a key component of rehabilitation but remains understudied and underspecified in research and practice, partly due to limited conceptual understanding and comprehensive assessment tools. This gap may hinder the integration of rest advice into physical activity and fatigue management protocols. This study aimed to develop and validate the Multidimensional Rest Experience Scale (MRES) for adults with Chronically Fatiguing Conditions (CFC).

**Methods:**

Conducted in three phases, Phase 1 involved MRES development via a modified Delphi method to ensure content validity. Phase 2 included preliminary validation by administering the MRES to a representative sample of adults with CFC to assess construct validity and factor structure. Phase 3 evaluated reliability, examining internal consistency and test-retest reliability. Descriptive and inferential analyses were performed.

**Results:**

The Delphi process reached consensus on 25 items. Exploratory factor analysis refined the scale to 22 items with six factors: resting activity, restorative rest, planning and prioritising rest, physical and psychological barriers to rest, emotional response to rest, and risk of under-resting. Resting Activity showed significant positive correlations with Chronic Pain Coping Inventory (CPCI) Rest subscale (r = 0.572, p < 0.001) and Pacing Engagement (r = 0.332, p < 0.001). Planning and Prioritising Rest correlated with CPCI-Rest (r = 0.532, p < 0.001) and Pacing Engagement (r = 0.681, p < 0.0001). Under-resting risk correlated with Over-Activity risk (r = 0.504, p < 0.001). Discriminant validity for the MRES-22 subscales was established, with Heterotrait-Monotrait (HTMT) ratios ≤ 0.85. Internal consistency was acceptable to good (Cronbach’s α = 0.773–0.829, CR = 0.752–0.830), and test-retest reliability was good to excellent (ICC = 0.816–0.943).

**Conclusion:**

The MRES-22 is a valid and reliable tool for capturing the multidimensional components of rest experience, which are often overlooked in fatigue management protocols. This finding supports the use of the MRES-22 in both clinical and research settings to enhance understanding of rest in relation to key health outcomes and to optimise fatigue interventions in rehabilitation.

## Introduction

Fatigue is a complex, enduring state of physical and mental exhaustion characterised by a profound lack of energy that is disproportionate to exertion and remains unrelieved by rest [1–4]. Fatigue is associated with reduced functional independence, increased mortality risk, and higher healthcare utilisation in adults with Chronically Fatiguing Conditions (CFC) such as stroke, rheumatological conditions, and cancer [5–8]. More importantly, clinical guidelines and evidence-based strategies for managing fatigue in these populations are sparse [9,10]. Consequently, promoting an active lifestyle alongside activity management strategies may be essential for alleviating fatigue and supporting daily functioning [11,12]. Activity pacing is a behavioural process that can be at the basis of promoting an active lifestyle by reducing fatigue and enhancing physical activity in adults with CFC [12]. Development of activity pacing interventions may help individuals with CFC manage symptoms, improve functional abilities, and maintain or even gradually increase physical activity [12–15]. The objectives include enhancing functional capacity, reducing symptom severity, promoting sustained physical activity, and improving overall daily functioning [12–15].

Activity pacing initially emphasised an energy conservation strategy and activity reduction, encouraging strict adherence to energy limits to prevent symptom exacerbation [16–18]. More recently, it has evolved to incorporate individualised behavioural patterns, providing tailored interventions that correspond with each person’s unique tendencies and activity levels [13,19]. For those inclined to avoiding activity due to fatigue for fear of relapse, strategies for graded and consistent activity are advised [13]. Conversely, individuals prone to over-activity during low-fatigue periods, leading to severe fatigue and prolonged inactivity, are guided to adopt a consistent activity-rest regimen, with gradual increases in activity [13,14,20]. Recent reviews and different study designs suggest that appropriate use of activity pacing may help reduce fatigue and promote increased physical activity among adults with CFC, though the evidence base remains inconclusive despite promising preliminary findings [13,18,21–24]. In addition to distributing activities throughout the day, adequate rest is a crucial component of interventions such as activity pacing, aiming to manage fatigue and optimise health outcomes for adults with CFC [13,24].

Rest is an important component for effectively managing fatigue and optimising activity levels in rehabilitation [25]. Recent scoping reviews and qualitative studies underscore the potential importance of rest for adults with CFC, highlighting benefits such as improved energy levels, enhanced activity management, and symptom reduction [26–28]. Other studies have documented positive effects of rest on physical function [29–31]. These findings have implications for fatigue management and may support the efficacy of interventions such as activity pacing, which emphasise anticipating fatigue and incorporating adequate rest. As the potential benefits of rest in rehabilitation for managing adults with CFC become more apparent, it is essential to further develop our conceptual understanding of rest especially regarding it quality and quantitative. In a recent study, a gap in literature has been highlighted, signalling that even though many interventions highlighted the importance of rest, there was no structured way of reporting and/or analysing rest in literature, which makes it difficult to make solid recommendations about optimal rest [25]. This may partly stem from a lack of consensus regarding the definition of rest within the context of rehabilitation [32] and the absence of standardised measures to assess its quality and quantity. As a result, the role of rest in managing fatigue and optimising rehabilitation outcomes remains underexplored, leaving Healthcare Professionals (HCP) and researchers with insufficient evidence to guide its integration into clinical practice. Additionally, and more importantly, our scoping review and qualitative findings suggest that future interventions should include systematic documentation of the type, quality and quantity of rest, as this may be essential for enhancing self-management strategies, particularly in the context of physical activity promotion [26–28]. However, implementing such recommendations may present challenges, as no comprehensive measures currently exist to assess rest-related constructs or guide rest advice in adults with CFC.

At present, there are relatively few validated scales available to holistically measure the concept of rest [33,34]. A notable limitation of existing tools is their tendency to focus predominantly on a single construct, which may not fully capture the complexity of rest as an experience [35]. Furthermore, the limitations of existing rest scales may be attributed to the lack of patient involvement during their development. Stakeholder input is crucial to ensure that the constructs and content are both relevant and reflective of target population’s lived experiences [36,37]. The absence of such involvement may result in scales that inadequately reflect the complexity of how rest is perceived and used. These limitations underscore the need for a new, comprehensive measure of rest that incorporates perspectives from both patients and HCP. Consequently, the study aims to develop and validate a rest measure, hereafter referred to as the Multidimensional Rest Scale (MRES) for adults with CFC. In this context, rest experience is operationally defined as the overall perception and subjective sense of restfulness, encompassing not only the physical cessation of activity but also the cognitive, emotional, and environmental/social dimensions that collectively contribute to the restorative quality derived from rest [26]. It reflects the extent to which rest effectively enhances energy levels, facilitates the performance of activities of daily living, and ultimately supports overall well-being and psychological functioning [26]

In light of the above, the specific objectives of the study were threefold: (1) to develop the Multidimensional Rest Experience Scale (MRES) for adults with CFC, (2) to validate the MRES within this target population to ensure its psychometric soundness, and (3) to establish the reliability of the scale to confirm its consistency in measuring rest experience among adults with CFC. To achieve these objectives, the study was systematically conducted in three sequential phases. The initial phase focused on the development of the MRES questionnaire, employing a modified Delphi method [38] to incorporate expert consensus and ensure content validity. The second phase involved the preliminary validation of the MRES by administering it to a representative sample of adults with CFC, allowing for assessment of its construct validity and factor structure. The third and final phase concentrated on evaluating the initial reliability of the MRES, examining the internal consistency and test-retest reliability within the same population. The overarching aim of developing the MRES is to enhance our understanding of the multifaceted nature of rest as experienced by adults with CFC, thereby contributing to the development of tailored interventions (e.g., activity pacing) to alleviate fatigue and improve daily functioning.

## Methods

### Phase 1: Development phase

#### Design.

This study is part of the broader project titled “Exploring and optimising rest to alleviate fatigue and improve physical activity in persons with chronically fatiguing conditions.” The study protocol, procedures, and materials were reviewed and approved by the Northumbria University Ethics Online System under approval number 3779 [reference Ackah 2023-3779-4110].

This study adhered to the modified guideline for Conducting and Reporting of Delphi Studies (CREDES) [39] and COSMIN (Consensus-based Standards for the selection of health Measurement Instruments) [40].The consensus-building process followed a three-step modified Delphi method [41]. The modified Delphi strategy is an iterative process, extensively employed in healthcare to establish a consensus on a clinical concept [42,43]. This method systematically incorporates a literature review, gathers opinions from stakeholders, and relies on expert judgments within a field to reach a consensus. Moreover, Delphi enables a diverse range of people, with various backgrounds, expertise, and from different geographical locations, to participate anonymously, preventing domination of the group by a few expert viewpoints [44]. Consequently, our approach involved reviewing relevant literature and conducting in-depth interviews with adults with CFC and HCP including physiotherapists, occupational therapists, and health psychologists involved in the management of persons with CFC. Additionally, we incorporated two rounds of Delphi to achieve consensus on each generated item. Moreover, the study adhered to the COSMIN guidelines to ensure a rigorous and standardised approach to the development, validation, and reliability assessment of the MRES [40].

#### Participants and recruitment.

In this study, the term ‘experts’ was defined to include two groups: adults with CFC and HCPs involved in managing adults with CFC. Adults with CFC were recruited through established patient organisations and support groups across the UK, including the Long Covid Support Group-UK, Sjögren’s Syndrome UK, Multiple Sclerosis UK, Fibromyalgia Support UK, and Versus Arthritis UK. To participate, adults with CFC needed to identify fatigue as a key symptom of their condition, with clinically significant fatigue defined as a score of 4 or above on the fatigue severity scale [45]. They also needed to have the capacity and motivation to take part in a research interview lasting between 20 and 60 minutes, be fluent in English to ensure meaningful participation, and be aged 18 years or older.

HCPs were recruited within the UK using professional networks and a snowball sampling approach, whereby existing contacts recommended other eligible professionals. These HCPs were required to have at least ten years of professional experience in the UK health care system and to be directly involved in the management of adults with CFC. HCPs often hold expertise in designing and implementing rehabilitation interventions and can elucidate how rest is operationalised within clinical guidelines, therapy plans, and self-management support. Their input is also valuable in identifying potential barriers and facilitators to incorporating rest within rehabilitation frameworks, based on their practical experience with diverse patient populations. Furthermore, including HCPs enhances the credibility and applicability of the study findings by ensuring that the conceptualisation of rest is not only patient-centred but also aligned with clinical feasibility.

### Item list generation process and consensus-building

#### Stage 1: Literature review and Qualitative interview.

The item generation process was drawn from two main sources: Firstly, we reviewed the existing literature to understand the concept of rest in peer-reviewed articles. The full methodology for the literature review including search strategy, eligibility criteria, and data synthesis has been published previously [26]. In brief, the main concept of interest was rest [26]. While sleep and rest are closely linked, they are distinct in their underlying mechanisms [46,47]. Therefore, this review concentrated on wakeful rest. Studies focusing specifically on bed rest, as well as those involving rest in people with concussion, were excluded. A broad range of study designs examining rest were considered, including qualitative and quantitative studies, reviews, and concept analyses. However, studies without accessible full texts, those investigating resting-state EEG, and research exploring rest outside the context of health and rehabilitation were excluded. Literature searches were carried out in PubMed, the Cumulative Index to Nursing and Allied Health Literature (CINAHL), and PsycINFO to investigate the concept of rest within rehabilitation and in a wider context. Searches were conducted from inception to September 2024, when the final search was completed [26]. Details of the study selection and screening process are presented in the PRISMA flow diagram (see Supplementary File 1).

Secondly, a semi-structured interview guide was developed to explore concept of rest from perspectives of adults with CFC and HCP. The interviews were carried out through Microsoft Teams and varied in duration, lasting anywhere from 20 to 52 minutes. Overall, a panel of 27 experts were involved in the qualitative interview stage (See supplementary file 2). The literature and qualitative interviews were used to draft initial items for the questionnaire, which consisted of 41 items. Content validity was assessed using modified Delphi methods to ensure the relevance and appropriateness of the items [44].

#### Stage 2: Consensus-building and content validation.

The study utilised a modified Delphi method [44] to reach a consensus on the selection of relevant questions from stage one for inclusion in the final questionnaire. This process involved two rounds of evaluations by expert panellists over a three-month duration, from January 2024 to March 2024. The questionnaire was developed and administered via JISC survey software.

**Fig 1:**
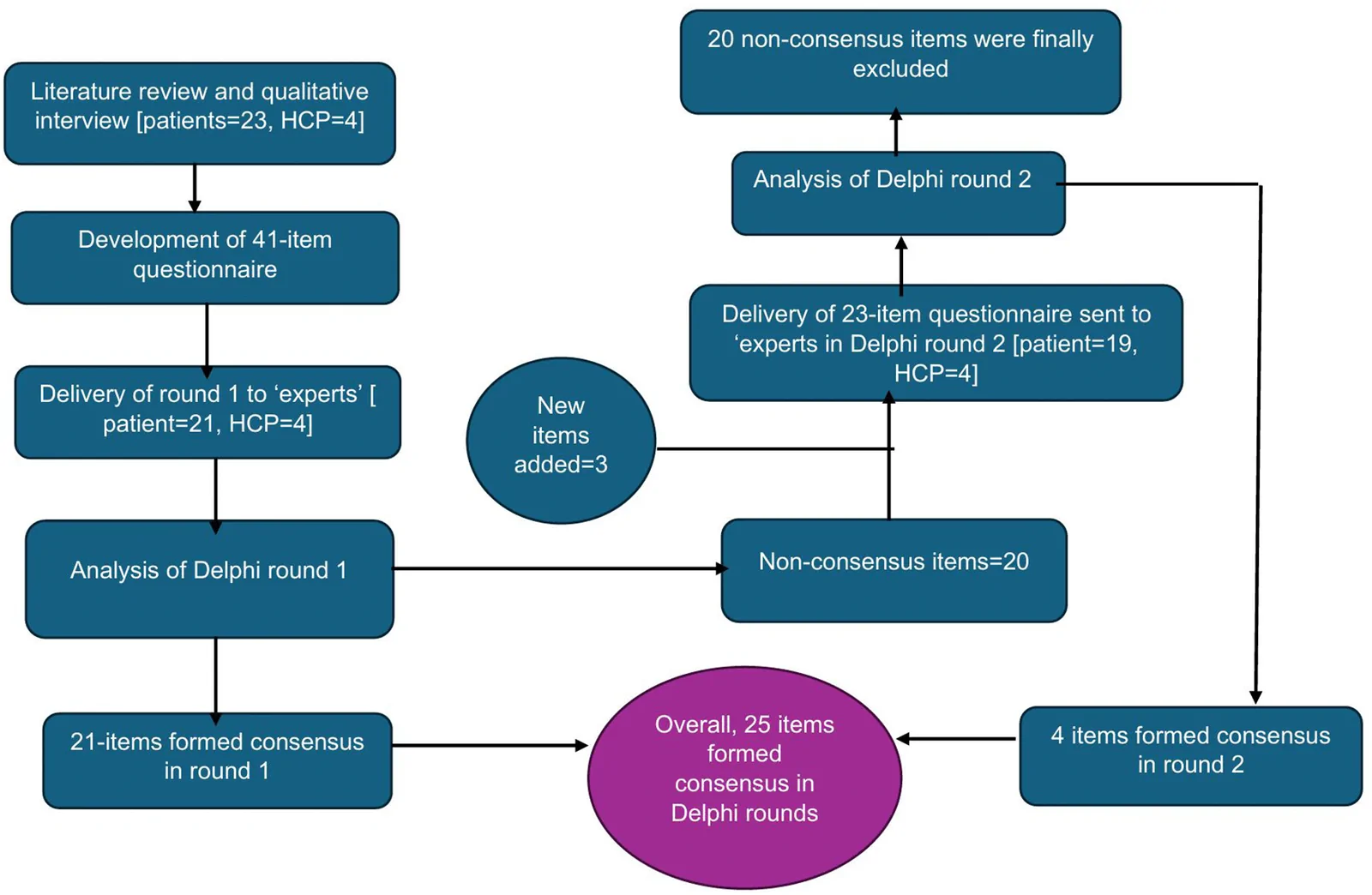
Flowchart of stages of the modified Delphi process.

Firstly, the Modified Delphi round 1 consisted of the 41-item structured statement/question identified through literature review and qualitative interview. The panels of experts were asked to rate the extent to which they agreed or disagreed with each item. This was done using a 5-point Likert scale, which included options for ‘strongly disagree,’ ‘disagree,’ ‘neither’ ‘agree,’ and ‘strongly agree. Anonymised results were downloaded from JISC and reviewed by the research team. Items which achieved consensus were retained to be included in the final questionnaire. In instances where experts did not reach consensus (i.e., < 70% agreement), we examined the comments provided in the open free-text section. These comments were carefully reviewed and discussed by the research team to guide the refinement of questionnaire items, following a process similar to that described by Antcliff et al. [48,49]. Based on this evaluation, some items were revised to improve clarity or framing, and three new items were added (Fig 1). The revised and new items were subsequently incorporated into the round 2 questionnaire.

Secondly, the non-consensus statements from round 1 were sent to the panel of experts in round 2. Like the first round, experts used the same voting method, but this time, they had knowledge of group scores. Therefore, participants had the opportunity to reconsider the group results and change their decision while maintaining the confidentiality of their responses. Final analyses of responses in round 2 followed the same criteria as in round 1. Statements not meeting expert consensus were excluded. Overall, this phase resulted in 25 items namely MRES-25.

### Phase 2: Validation stage

After developing the MRES, the next phase was to validate it in a representative sample of adults with CFC. To achieve this, a cross-sectional survey targeting a sample of adults CFC was caried out between April 20, 2024, and August 19, 2024. Participants in the survey phase were adults with CFC. Inclusion criteria required participants to be aged 18 years or older, fluent in English, and to score ≥4 on the Fatigue Severity Scale [45]. Exclusion criteria included adults with chronic conditions who did not experience fatigue, individuals under 18 years of age, severely ill persons, and non-native English speakers. Participants were recruited through patient organisations and support groups across the UK, including the Long Covid Support Group-UK, Sjögren’s Syndrome UK, Multiple Sclerosis UK, Fibromyalgia Support UK, and Versus Arthritis UK. The sample size was determined based on widely accepted guidelines which recommend that the sample size should be at least five to ten times the number of items included in the scale [50–54]. The MRES used in this study consists of 25 items. Applying the lower limit of this guideline, which suggests five participants per item, results in a required sample size of 125 participants (5 participants × 25 items). Consequently, adhering to this standard, a minimum of 125 participants was considered adequate for this phase of the research.

In addition to completing the MRES, participants were also asked to complete measures assessing theoretically similar constructs identified in the literature. These included the Rest subscale of the CPCI [33], which assesses rest as a coping strategy in chronic conditions, and the Activity Pacing and Risk of Over-Activity Questionnaire, which was developed for the ReSpAct study [55]. The inclusion of these instruments aimed to facilitate the assessment of convergent validity for the MRES by examining the extent to which it correlates with related constructs, thereby providing further evidence of its validity and potential utility within clinical and rehabilitation settings. Data were collected and administered via JISC online survey software.

### Phase 3: Reliability

In Phase 3, test-retest reliability was assessed to determine the temporal stability of the MRES scores. All participants who completed the validation stage (Time 1) were invited to complete the same questionnaire again two weeks later (Time 2). Only participants who completed the questionnaire at both time points were included in the test-retest reliability analysis. A two-week interval was chosen as it is widely recommended for assessing test-retest reliability. This duration is considered sufficient to minimise the possibility of participants recalling their previous responses while ensuring that the construct measured remains stable [53,56], thus avoiding genuine changes that may occur over longer periods. Given that the experience of rest, as conceptualised in this study, is expected to remain relatively stable in the absence of major life events or interventions, a two-week interval was deemed appropriate to determine the stability of the measure over time [53,56].

### Data analysis

#### Phase 1 analysis.

Phase 1 data were analysed using descriptive statistics. Demographic information from modified Delphi round 1 was used as a baseline to describe the characteristics of the participants. This included sex distributions and types of participants. Frequency and percentages were used to estimate and describe each round of the Delphi process. For consensus items, responses of “strongly agree” and “agree” were combined. Consensus for inclusion was defined a priori as ≥70% of participants strongly agreeing or agreeing that an item should be included in the questionnaire, consistent with established consensus thresholds in Delphi studies [49,57,58]. Items that did not meet this criterion, including those with proportions of “strongly disagree,” “disagree,” or “neutral” responses, were classified as non-consensus items. Fisher’s exact test was used to show the difference between the type of participants (adults with CFC vs HCP) with regards to their responses.

#### Phase 2 analysis.

An Exploratory Factor Analysis (EFA) was first conducted to identify the underlying dimensions of the scale, resulting in six distinct factors. Partial Least Squares Structural Equation Modelling (PLS-SEM) with bootstrapping was then used to assess the measurement model, with each dimension modelled as a reflective first-order construct, consistent with reflective measurement theory, where items were conceptualised as manifestations of their respective latent dimension. Given the lack of empirical support for modelling the dimensions as a higher-order formative or reflective construct, the MRES is conceptualised as a multidimensional tool assessing distinct yet related aspects of rest experience. Consequently, each facet of the MRES was modelled as a reflective construct to enable a comprehensive evaluation of its psychometric properties. Specifically, indicator reliability was assessed through examination of the outer loadings to ensure that each item adequately represented its underlying dimension. Internal consistency was evaluated using Cronbach’s alpha, while composite reliability was calculated to provide an additional measure of scale reliability. Convergent validity was assessed by calculating the Average Variance Extracted (AVE) for each dimension, with values of 0.50 or higher indicating adequate convergent validity [59]. In addition to convergent validity, the subscales were correlated with the pacing engagement and rest subscales of the CPCI using Spearman correlation. Finally, discriminant validity was evaluated using the Heterotrait-Monotrait (HTMT) ratio of correlations (<0.85) to ensure that each construct was distinct from the others within the model [59].

#### Phase 3 analysis.

A two-way mixed-effects model with absolute agreement was employed to evaluate test-retest reliability, producing Intraclass Correlation Coefficient (ICC) estimates to assess the stability of the MRES over time. Additionally, measurement error indices were calculated, including the Standard Error of Measurement (SEM), computed as SEM = SD × √ (1 − reliability coefficient) [60], and the Minimal Detectable Change (MDC), calculated as MDC = SEM × z-value × √2 [61]. The SEM quantifies the precision of individual scores by estimating the margin of error around observed scores, while the MDC represents the smallest detectable change that exceeds measurement error, indicating a true change rather than mere measurement variability.

Overall, all quantitative analyses were conducted using SPSS software (version 29) and SmartPLS 4, with Microsoft Office Excel 2013 utilised for data management and organisation.

## Results

### Phases 1 results: Delphi stage

#### Characteristics of delphi participants, content validity, and consensus items.

A total of 27 participants were involved, which included 23 adults with CFC (long covid, ME/CFS, arthritis, multiple sclerosis) and four HCP (two occupational therapists, one physiotherapist, and one health psychologist) (See supplementary file 2). The HCP had between 14 and 30 years of experience managing adults with CFC at the time of data collection. Among these participants, 17 were female, accounting for 68% of the group. 25 participants transitioned to the first round of the Delphi process. This group consisted of 21 adults with CFC and four HCP, maintaining the same representation of HCP. The Delphi process continued with strong engagement, as 23 participants completed the final round. This included 18 adults with CFC and the same four HCP. The overall response rates were high, with 25 out of 27 participants (92.5%) completing the first round and 23 out of 25 participants (92%) completing the second round.

In the first round of the Delphi process, a total of 41 items were distributed to the participants for evaluation. Following the participants’ reviews and votes, 21 of these items were found to be important and were included in MRES. Additionally, feedback from the participants during this round led to the introduction of three new items that were not part of the original set but were considered important based on their comments. The revised list, which included the items with non-consensus from round 1 as well as the three newly added items, was then sent to the same group of participants for the second round of evaluation. During this second round, consensus was reached on four of the new extra items. Consequently, a total of 25 items, which achieved consensus across both rounds of the Delphi process, were finalised and included in the final version of the MRES-25. (See Fig 1).

Fisher’s exact test was employed to assess whether there were significant differences in responses between adults with CFC and HCP concerning the final consensus items. The results of this analysis indicated that there were no statistically significant differences between the responses of these two groups (See Table 1).

**Table 1:** Final consensus item included in the MRES and Fisher’s exact results.

MRES-25 items	Consensus [%]	Fisher’s exact test
1. My rest involves reducing physical activity	76%	ns
2. My rest involves reducing mental activity.	80%	ns
3. My rest involves stopping my activity	72%	ns
4. My rest involves sleeping	70%	ns
5. My rest involves detaching from my core activity	74%	ns
6. My rest involves reducing sensory stimulation (e.g., noise, light)	100%	–
7. My rest involves engaging in relaxation activity (e.g., meditation)	88%	ns
8. My rest involves sitting/lying down	76%	ns
9. Rest recharges my energy	80%	ns
10. Rest helps me manage my fatigue.	84%	ns
11. Rest helps me perform my daily activities	84%	ns
12. Rest helps me improve my well-being	76%	ns
13. I find it difficult to rest during the day	88%	ns
14. Stress makes it difficult for me to rest	92%	ns
15. Anxiety makes it difficult for me to rest	88%	ns
16. Pain makes it difficult for me to rest	96%	ns
17. I find it difficult to stop and rest	76%	ns
18. Racing thoughts prevent me from resting better	76%	ns
19. I rest better in serene environment	83%	ns
20. I feel guilty when I stop to rest.	88%	ns
21. I feel frustrated when I rest	76%	ns
22. I take short breaks from activities	83%	ns
23. I prioritise self-care, including periods of rest.	84%	ns
24. I plan specific periods of rest.	88%	ns
25. I schedule regular rest breaks throughout the day	72%	ns

SD=Strongly Disagree, D=Disagree, N=neutral, A=Agree, SA=Strongly Agree, ns=non-significant.

### Phase 2 results: Validation stage

#### Characteristics of the participants.

The study sample included 162 participants with a mean age of 49.8 years (±12.6), of whom 80.9% were female. The majority were married (55.6%) and employed (42.0%). The most common primary diagnoses were Long COVID (24.1%). Participants had experienced fatigue for an average of 5.62 years (±0.62). The missing values for the questionnaire items ranged from 0.6% to 1.8%. Further demographics are detailed in Table 2.

**Table 2:** Characteristics of the participants at the survey stage.

Variable	Frequency (%) or mean± SD, n = 162
Age (in years)	49.8 ± 12.6
Number of females	131 (80.9)
**Marital status**	
Single	31 (19.1)
Married	90 (55.6)
Divorce	19 (11.7)
Co-habiting	16 (9.9)
Widow/widow	6 (3.7)
**Employment status**	
Working	68 (42.0)
Not working	65 (40.1)
Retired	29 (17.9)
**Ethnic group**	
White	156 (96.3)
Black/others	6 (3.7)
**Primary diagnosis**	
Long covid	39 (24.1)
ME/CFS	32 (19.8)
Arthritis (OA/RA)	30 (18.5)
Multiple sclerosis	29 (17.9)
Stroke	32 (19.8)
Fatigue experience (in years)	5.62 ± 0.62

OA=osteoarthritis, RA=Rheumatoid arthritis, ME/CFS= Myalgic Encephalomyelitis/Chronic Fatigue Syndrome.

#### Exploratory factor analysis.

The strength of the relationship among the items was tested using the coefficient of correlation. The results showed that there was evidence of a coefficient of correlation greater than 0.30 in the matrix. The presence of multicollinearity was tested using the determinant score. The results showed that the determinant score was less than 0.000001, suggesting an absence of multicollinearity. Additionally, the results of the Kaiser-Meyer-Olkin measure was 73.4%, and Bartlett’s test of sphericity was significant (p < 0.001) for all the items. This suggests that the data were suitable for factor analysis.

The initial unrotated component extraction for the MRES-25 revealed seven components with Eigenvalues >1, explaining 66.3% of the variance. Following the component extraction, the correlation matrix was carefully inspected to evaluate the relationships between the individual items. During this inspection, one item was identified as having poor correlations and was removed (item4: “My rest involves sleeping”).

Exploratory factor analysis with Oblimin rotation and Kaiser normalisation was conducted on the remaining 24 items. Following this analysis, two items were excluded due to inadequate factor loadings (i.e., loadings <0.40) and significant cross-loadings (i.e., loadings >1). Specifically, Item 7 (“My rest involves engaging in relaxation activities”) and Item 19 (“I rest better in a serene environment”) were removed.

**Fig 2:**
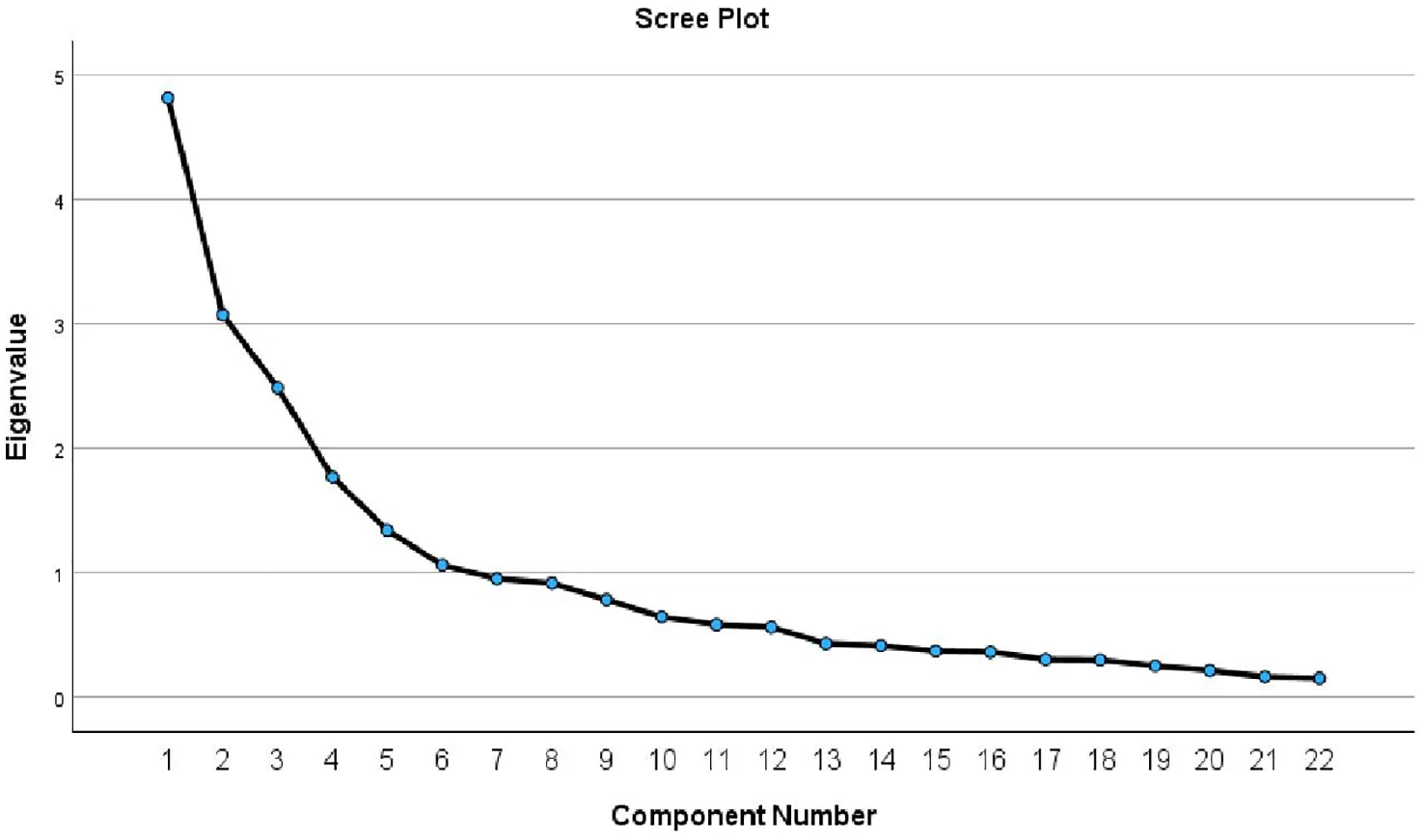
Unrotated Scree plot showing six components before extraction with eigenvalues>1.

The analysis was re-conducted on the remaining 22 items, yielding six components with Eigenvalues greater than 1 (see Fig 2), which collectively accounted for 66.2% of the total variance. At this stage, no items were removed, as all communalities exceeded 0.30, and no instances of multiple cross-loadings were observed (Table 3).

**Table 3:** Six-factor solution for the MRES using principal component with Oblimin rotation.

MRES sub-scale	Factor Loading (Pattern matrix)	Communality	Mean ±SD
**Factor 1: Rest activity**			
Q5: My rest involves detaching from my core activity	0.731	0.616	4.10 ± 0.88
Q2: My rest involves reducing mental activity	0.721	0.622	4.20 ± 0.90
Q3: My rest involves stopping my activity	0.719	0.704	4.06 ± 0.98
Q1: My rest involves reducing physical activity	0.662	0.515	4.75 ± 0.62
Q8: My rest involves sitting/lying down	0.589	0.393	4.80 ± 0.44
Q6: My rest involves reducing sensory stimulations (e.g., noise, light)	0.553	0.582	3.95 ± 1.07
**Factor 2: Restorative Rest**			
Q10: Rest helps me manage my fatigue	0.819	0.704	3.49 ± 1.00
Q12: Rest helps me improve my well-being	0.818	0.700	3.29 ± 0.97
Q11: Rest helps me perform my daily activities	0.800	0.662	3.28 ± 1.05
Q9: Rest recharges my energy	0.749	0.610	2.81 ± 0.95
**Factor 3: Planning and prioritisation of rest**			
Q24: I plan specific periods of rest	0.919	0.814	3.88 ± 1.02
Q25: I schedule regular rest breaks throughout the day	0.890	0.820	3.30 ± 1.25
Q22: I take short break from activities	0.570	0.775	3.82 ± 0.96
Q23: I prioritise self-care including periods of rest	0.562	0.624	3.48 ± 1.20
**Factor 4: Physical and psychological barriers to rest**			
Q15: Anxiety makes it difficult for me to rest	0.847	0.784	2.80 ± 1.23
Q14: Stress makes it difficult for me to rest	0.713	0.765	3.17 ± 1.19
Q18: Racing thoughts preventing me from resting better	0.685	0.581	2.99 ± 1.10
Q16: Pain makes it difficult for me to rest	0.478	0.423	2.94 ± 1.24
**Factor 5: Emotional response**			
Q21: I feel frustrated when I rest	0.850	0.775	3.54 ± 1.14
Q20: I feel guilty when I stop to rest.	0.827	0.732	3.21 ± 1.31
**Factor 6: Risk of under-resting/lack of skill**			
Q13: I find it difficult to rest during the day	0.823	0.775	2.94 ± 1.11
Q17: I find it difficult to stop and rest	0.734	0.748	2.99 ± 1.07

Q=Question, SD=Standard Deviation.

### Dimensional structure and validity components of MRES-22 via partial least squares structural equation modelling

#### Indicator reliability.

Indicator reliability, assessed via outer loadings of individual items on their respective constructs, exhibited some variation. The lowest outer loading was observed for item Q8 (0.486) within the resting activity dimension. Conversely, the highest loadings were observed for items Q13 and Q17 (both 0.917) within the lack of skill to rest dimension. Most other items demonstrated robust loadings exceeding 0.60. These findings are presented in Table 4.

**Table 4:** Outer Loadings, Cronbach’s Alpha, Composite Reliability, and HTMT for the MRES-22 Constructs (PLS-SEM Analysis).

Construct	item	Outer loading	Cronbach’s α	CR	AVE	HMTM
Resting activity	Q1	0.576	0.773	0.799	0.471	Yes
	Q2	0.767				
	Q3	0.818				
	Q5	0.778				
	Q6	0.661				
	Q8	0.486				
Restorative rest	Q9	0.771	0.829	0.830	0.661	Yes
	Q10	0.824				
	Q11	0.826				
	Q12	0.829				
Physical-psychological barriers	Q14	0.869	0.765	0.812		Yes
	Q15	0.893				
	Q16	0.516				
	Q18	0.766				
Lack of skill to rest	Q13	0.917	0.811	0.811	0.841	Yes
	Q17	0.917				
Emotional response to rest	Q20	0.895	0.752	0.752	0.801	Yes
	Q21	0.895				
Planning and prioritising rest	Q22	0.602	0.792	0.820	0.625	Yes
	Q23	0.771				
	Q24	0.876				
	Q25	0.881				

CR = Composite Reliability, AVE = Average Variance Extracted, HTMT = Heterotrait-Monotrait ratio

#### Convergent validity.

Convergent validity, as measured by the AVE ranged from 0.471 for the resting activity construct to 0.841 for the Lack of Skill to Rest construct. While most constructs exceeded the standard AVE threshold of 0.50, the Resting Activity construct’s AVE was marginally below this criterion, indicating somewhat limited shared variance among its items. Despite this, the construct was retained due to other supporting reliability metrics. Table 4 presents these findings.

Additionally, the associations between the subscales of the MRES-22 and the validated subscales of the CPCI, including the rest subscale and pacing engagement were examined to evaluate their relationships and convergent validity. The Resting Activity subscale from the MRES-22 demonstrated a significant positive correlation with the Rest subscale of the CPCI (r = 0.572, p < 0.001). Additionally, the Resting Activity subscale was positively correlated with Pacing Engagement (r = 0.332, p < 0.001). The planning and prioritising rest subscale was positively correlated with the Rest subscale of the CPCI (r = 0.532, p < 0.001). Additionally, the Planning and Prioritising Rest subscale exhibited a substantial positive correlation with Pacing Engagement (r = 0.681, p < 0.0001). The risk of under-resting component of the MRES-22 was evaluated in relation to the Risk of Over-Activity. The analysis revealed a significant positive relationship between these two variables, with a correlation coefficient of (r = 0.504, p < 0.001).

#### Discriminant validity.

Discriminant validity of the MRES-22 subscales was assessed using the HTMT criterion, with results presented in supplementary file 3. HTMT values ranged from 0.098 between Lack of Skill to Rest and Restorative Rest to 0.542 between Lack of Skill to Rest and Physical and Psychological Barriers to Rest. The Emotional Response to Rest subscale showed low to moderate correlations with other constructs (HTMT values ranging from 0.147 to 0.47). Planning and prioritising rest demonstrated moderate discriminant validity with Lack of Skill to Rest (0.515) and Emotional Response to Rest (0.47), while its relationship with Physical and Psychological Barriers to Rest (0.364) remained below the threshold, indicating differentiation between these constructs. Furthermore, Resting Activity and Restorative Rest displayed low HTMT values with all other subscales (ranging from 0.098 to 0.425). The results are shown in Table 4.

### Phase 3 results: Reliability stage

#### Internal consistency.

The internal consistency of the MRES-22 was strong, as indicated by Cronbach’s alpha values for its subscales: Lack of Skill to Rest (α = 0.811), Restorative Rest (α = 0.829), Resting Activity (α = 0.773), Planning and Prioritising Rest (α = 0.792), Emotional Response to Rest (α = 0.752), and Physical-Psychological Barriers (α = 0.765). Additionally, composite reliability was acceptable for Lack of Skill to Rest (CR = 0.799), Restorative Rest (CR = 0.830), Resting Activity (CR = 0.799), and Physical-Psychological Barriers (CR = 0.812). (See Table 4)

#### Test-retest reliability.

The results of the ICC analysis for the MRES-22 sub-scales indicate a high level of reliability across repeated measurements: The results of the ICC for the resting activity sub-scale were ICC = 0.828 [95% CI: 0.731–0.890]. The restorative rest sub-scale had an ICC of 0.816 [95% CI: 0.711–0.883]. Similarly, the planning and prioritising rest sub-scale had an ICC of 0.861 [95% CI: 0.781–0.912]. The ICC values for the remaining MRES-22 subscales, as well as the SEM and MDC, are detailed in Table 5.

**Table 5:** Intra-class corelation coefficient based on absolute agreement and two-way mixed effects model.

MRES-subscales	Intraclass corelation	95% Confidence Interval		p-value	SD	SEM	MDC
		Lower bound	Upper bound				
Resting activity	0.828	0.731	0.890	<0.001	3.670	1.523	4.221
Restorative Rest	0.848	0.761	0.904	<0.001	3.273	1.276	3.537
Planning and prioritising rest	0.861	0.781	0.912	<0.001	3.667	1.367	3.789
Physical and psychological barriers to rest	0.943	0.911	0.964	<0.001	3.566	0.851	2.359
Emotional response to rest	0.871	0.798	0.918	<0.001	2.061	0.740	2.051
Risk of under-resting/lack of skill	0.860	0.781	0.910	<0.001	2.133	0.798	2.211

MDC=Minimal Detectable Change, SD= Standard Deviation, SEM=Standard Error of Measurement.

#### Scoring methods.

The instrument comprises six dimensions designed to capture various facets of the experience of rest: resting activity, restorative rest, physical and psychological barriers, planning and prioritising rest, and lack of skill. Each dimension consists of multiple items rated on a five-point Likert scale with response categories ranging from Never (1), Rarely (2), Sometimes (3), Often (4), to Very Often (5).

For scoring purposes, each item response is assigned a corresponding numerical value from 1 to 5. Dimension scores are computed by calculating the arithmetic mean of the item scores within each dimension, thereby preserving the original measurement scale and facilitating interpretability.

Given the empirical evidence indicating that the six dimensions represent distinct yet related constructs rather than indicators of a single higher-order factor, composite scores are reported separately for each dimension. This multidimensional conceptualisation enables a comprehensive assessment of the participants rest experience across multiple domains.

Higher scores on each dimension indicate a more frequent occurrence of the aspect of rest experience. For instance, a higher score on the restorative rest dimension suggests better quality of restorative rest, while a higher score on physical barriers indicates greater perceived obstacles to resting

This scoring approach is consistent with the reflective measurement model established during the validation process and supports the nuanced interpretation of the scale’s multidimensional structure.

## Discussion

Rest is an important yet understudied and poorly defined component of rehabilitation interventions, limiting its integration into physical activity and fatigue management protocols and hindering the optimisation of rest strategies within activity pacing interventions. This study addressed this gap by developing the MRES with input from adults with CFC and HCPs. Through a Delphi process, 25 consensus items were identified and refined to 22 items via exploratory factor analysis, resulting in six components: resting activity, restorative rest, planning and prioritising rest, barriers to rest, emotional response to rest, and risks of under-resting. A first-order reflective model was adopted, conceptualising the MRES-22 as a multidimensional reflective measure, with each dimension assessing a distinct facet of rest and collectively capturing the broader construct of rest experience among adults with CFC and fatigue. The subscales demonstrated good validity and reliability metrics, supporting its use in clinical and research settings to better understand rest and optimise fatigue interventions in rehabilitation.

The first component of the MRES-22 is termed resting activity, which encompasses six items centred on the reduction or cessation of both physical and mental activities. This component also emphasises disengagement from primary activities and creating a calm, low-stimulation environment. This construct corroborates with the existing rest sub-scale found within the CPCI and other pain coping inventories [33,34,62], suggesting a conceptual consistency and underpinned by the energy conservation theory [63]. An important finding was that sleep formed consensus in the second round of the Delphi process. However, subsequent quantitative analysis indicated that this construct/item poorly correlated with other MRES items, resulting in its removal from the final instrument. Previous research has emphasised that sleep and rest are conceptually distinct phenomena [46,47]. Sleep is identified as a unique state of loss of consciousness with specific restorative functions, whereas rest is characterised by the interruption of activity, leading to a state of relaxation [46,64,65]. Overall, The MRES contains more items and subscales than the rest subscale of the CPCI and the AMI-P. While the latter scales have five and seven items respectively focusing on rest [33], [34], the MRES includes 22 items with the potential to provide a more comprehensive assessment of rest in adults with CFC to optimise interventions in rehabilitations.

The MRES identified a component labelled restorative rest. This sub-scale is particularly novel, as previous empirical rest-related measures have not highlighted the restorative component of rest in rehabilitation. This sub-scale may also underscore the quality of rest, suggesting that optimal rest could yield positive health outcomes, contrasting with the predominantly negative framing of rest in pain literature. Within pain management research, rest is frequently depicted as a passive behaviour, often viewed as detrimental due to its associations with avoidance, inactivity, and functional decline [18,66–68]. However, this sub-scale reorients this perspective, proposing that rest, when purposefully and optimally achieved, serves as an active and beneficial component of the restorative process. Specifically, optimal rest may contribute to reduced symptom severity, enhanced daily functioning, and improved overall patient well-being [69,70]. Indeed, to fully harness the benefits of rest, it should aim beyond mere inactivity to achieve outcomes like reduced fatigue, improved daily functioning, and enhanced well-being. This is consistent with Kaplan’s Attention Restoration Theory, which suggests that restorative activities, particularly being in nature, may promote cognitive function, alleviate mental fatigue, and improve well-being [69,71,72]. These positive outcomes of rest were also evident in our previous literature review and qualitative study [26,27]. Additionally, the MRES-22 features a component on planning and prioritising rest, which underscores the potential of strategically scheduling rest periods to optimise energy management and balance with activity. This finding corroborates with the principles of activity pacing [12–14]. Moreover, the MRES-22 introduces novel components that address physical and psychological barriers to rest, emotional responses to it, and the risk of under-resting. These components are particularly novel as they highlight previously underexplored dimensions of rest and fatigue management and may provide valuable insights for informing future research and interventions. Moreover, these findings align with the recently proposed multi-dimensional model of activity pacing for chronic pain and fatigue management [19]. Other components of the MRES-22 include physical and psychological barriers to rest, which represent external and internal obstacles, such as pain or stress, that can limit an individual’s ability to rest effectively despite their intentions. emotional response to rest reflects the affective dimension, highlighting how emotions such as relaxation, guilt, or frustration can either facilitate or inhibit rest behaviours. Finally, the risk of under-resting/lack of skill to rest dimension identifies adults with CFC vulnerability to insufficient rest due to factors like lifestyle demands, personal beliefs, or cultural pressures. Together, these dimensions illustrate that rest is not a simple behaviour, but a complex experience shaped by multiple factors, emphasising the importance of addressing each aspect in both research and clinical practice.

The relationships between the subscales of the MRES-22 and those of other validated measures provide strong and conceptually coherent evidence for its convergent validity. Specifically, the resting activity subscale of the MRES-22 showed significant positive correlations with both the rest subscale of the CPCI and the pacing engagement scale. This suggest that individuals who report greater engagement in resting behaviours on the MRES-22 also tend to report higher levels of rest on CPCI rest [33] and pacing engagement scales [55]. Similarly, the planning and prioritising rest subscale was also positively correlated with these measures. Additionally, the risk of under-resting subscale was positively associated with the risk of over-activity, which aligns with theoretical expectations. These correlations support the convergent validity of the MRES-22 by demonstrating meaningful links with the validated measures of rest and pacing [33,55]. These findings are consistent with principles in the rehabilitation of CFC where rest is understood to be an integral part of activity pacing; an approach designed to balance activity levels and minimise symptom exacerbation [55]. Therefore, a positive relationship between rest and pacing was anticipated. Likewise, the observed association between under-resting and over-activity reflects the common tendency for adults with CFC who struggle to rest adequately to overexert themselves, increasing their risk of fatigue and symptom flare-ups.

Further evidence for convergent validity was provided by the AVE. All subscales, with the exception of the ‘resting activity’ construct, exceeded the recommended threshold of 0.50, indicating that they account for more than half of the variance in their respective indicators [59]. This suggests that the items within each subscale are strongly related to their underlying construct. Although the resting activity subscale fell marginally below this threshold, it was retained due to its acceptable internal consistency and strong theoretical and content validity. In addition to convergent validity, the MRES-22 demonstrated good discriminant validity (e.g., as assessed using HTMT). This is important because it confirms that each subscale captures a unique dimension of the rest experience rather than redundant or overlapping constructs. From a practical perspective, this distinction enhances the interpretability of the scale, allowing clinicians and researchers to identify specific facet/domain of rest persons with CFC struggle with and to tailor interventions accordingly. Taken together, these findings provide a comprehensive picture of the validity of the MRES-22. The consistent pattern of theoretically aligned correlations, supported by both AVE and discriminant validity analyses, suggests that the scale is not only psychometrically sound but also meaningful in capturing real-world rest behaviours. As such, the MRES-22 may represent a credible, multidimensional tool for assessing diverse facets of rest-related perceptions, behaviours, and barriers. Its demonstrated validity enhances its potential utility in both research and clinical rehabilitation settings, where it may be used to inform targeted interventions, monitor patient progress, and improve understanding of rest-related outcomes in persons with CFC.

The findings presented indicate that the MRES-22 demonstrates both high reliability and stability over time, supporting its utility as a robust psychometric tool. Internal consistency, as evaluated through Cronbach’s alpha and composite reliability suggests that items within each sub-scale consistently measure their intended interrelated constructs. High Cronbach’s alpha and composite reliability values indicate that the items are homogenous in capturing constructs related to rest experience, thereby reinforcing confidence in the scale’s internal structure. Furthermore, test-retest reliability was assessed to determine the stability of the MRES-22 across time points. The intraclass correlation coefficient values obtained suggest that the measure yields consistent results upon repeated administrations, indicating minimal variation in responses over time. This is a crucial property for any measure intended to track changes in attitudes or behaviours longitudinally, as it demonstrates that any observed changes are more likely due to true differences rather than measurement error. Additionally, for clinical and research applications, the standard error of measurement and minimal detectable change were estimated. These parameters provide important indicators of the precision of the scale and the smallest change that can be interpreted as a real difference beyond measurement error, enhancing its interpretability in intervention or monitoring contexts. Overall, the combined evidence of strong internal consistency, high test-retest reliability, and clear clinical interpretability metrics suggests that the MRES-22 is a reliable and stable tool for assessing rest-related constructs across different time points and settings.

Taken together, the development and validation of the MRES-22 have important implications for enhancing activity pacing and thereby activity management in rehabilitation, particularly for adults with CFC. Activity pacing, which optimise function, is a promising component of rehabilitation strategies [12]. Now, most physical activity promotion studies and guidelines emphasise physical activity [73–75], while the equally important bouts of rest are less specified in rehabilitation [25]. Literature suggests that the quality and distribution of both rest and activity periods may be critical factors for the success of these interventions to maximise health benefits [26–28].The MRES-22 may provide a structured and comprehensive measurement tool to assess rest-related attitudes, emotions and behaviours, enabling clinicians to better understand each patient’s unique rest patterns, barriers, and needs. One major implication is that the MRES-22 detailed sub-scales, such as resting activity, restorative rest, and planning and prioritising rest, allow for a nuanced assessment of how patients incorporate and optimise rest. This detailed insight may enable HCPs to develop personalised pacing strategies that integrate these rest behaviours more optimally. Uniquely, the MRES-22 identifies specific barriers to rest both physical and psychological, and it identifies emotional responses to rest, both of which could be crucial to difficulties with resting, and both of which are often overlooked in traditional pacing strategies. By identifying these factors, clinicians can address the underlying obstacles that may hinder optimal pacing, such as emotional resistance to rest or physical discomfort. This may allow for more targeted interventions that could include specific cognitive-behavioural strategies and/or supportive resources to target these attitudes, emotions and barriers. Furthermore, the MRES-22 demonstrated reliability, and validity suggests that it can be used consistently over time to monitor patients’ rest and pacing behaviours as they progress through rehabilitation. This capability allows practitioners to adjust pacing recommendations dynamically, providing feedback that aligns with changes in the patient’s health status or functional capacity.

### Limitations, strengths and future directions

The study has limitations, warranting cautious interpretation of findings. Firstly, the demographic profile of the participants predominantly comprised people of White British ethnicity, with only 3.7% identifying as non-White British. Cultural, social, and structural factors can influence how fatigue is experienced, interpreted, and managed, including attitudes towards rest [76], expectations around activity, and access to healthcare resources. As such, the conceptualisation of rest captured in this study may be shaped by a relatively homogeneous cultural perspective, potentially overlooking variations in rest practices and meanings across ethnically diverse populations. Nonetheless, this underrepresentation of non-White British individuals is consistent with trends observed in most medical research conducted in the UK [77].

In addition, recruitment through patient support groups may have further contributed to sampling bias. Individuals who participate in such groups are often more engaged in managing their condition, may have greater access to information and peer support, and may be more motivated to reflect on and adapt their behaviours. They may also differ in terms of illness severity or duration, for example being more likely to seek support due to more persistent or impactful symptoms [78]. Consequently, the sample may overrepresent adults with CFC who are already actively engaged in self-management and underrepresent those who are less connected to support networks, newly diagnosed, or less able to engage due to socioeconomic or health-related barriers [78]. This may limit the representativeness of the findings and may skew the understanding of rest towards the experiences of a more health-engaged [78] subgroup of CFC.

Moreover, there was also a marked gender imbalance within the sample. Male participants were underrepresented overall, with all HCPs involved in the Delphi stage being female and only 25% of survey respondents identifying as male. This imbalance may have influenced the consensus process and the resulting conceptualisation of rest, as gender can shape both the experience and expression of fatigue, as well as attitudes towards rest and help-seeking. It important to highlight that societal norms may lead men to prioritise activity over rest, potentially resulting in different rest behaviours and needs that may not fully captured in the current study.

Furthermore, the responsiveness of the MRES-22 to detect meaningful change over time could not be assessed due to the lack of an intervention or event expected to alter participants’ rest-related behaviours or attitudes. Similarly, cross-cultural validity was not examined, limiting the generalisability of these findings beyond the current sample.

Despite these limitations, this study represents the first to incorporate both patients and HCP in the development of a MRES-22 in rehabilitation for adults with CFC. Furthermore, the use of the Delphi method in our study was instrumental in reducing the influence of dominant expert opinions. This methodological choice helps prevent any single expert’s views from disproportionately swaying the results, thereby enhancing the overall credibility of the item ratings. Our findings, supported by Fischer analysis, further substantiate the robustness of this approach. The Fischer analysis revealed no significant differences between the responses of the patient group and the HCP group, indicating that the consensus achieved through the Delphi method was representative of both perspectives. The over 90% response rate in both Delphi stages may have reduced potential bias and contributed to rigour and meaningful consensus [49,79]. Additionally, the robust statistical analyses employed to establish the reliability and validity of the tool represented a significant strength of this study. The questionnaire demonstrated strong feasibility, as evidenced by the low proportion of missing responses for individual items, which ranged between 0.6% and 1.8%. Such low levels of missing data indicate that participants found the items clear and manageable, reflecting the instrument’s practicality and ease of administration within the study population.

In light of these limitations, we recommend that future research employ longitudinal designs incorporating interventions or naturally occurring changes to evaluate the responsiveness of the MRES-22. Additionally, studies should assess its cross-cultural validity to ensure broader applicability across diverse populations and settings. Importantly, building on the findings of this study and recognising the role of optimal rest in managing fatigue and physical activity, future research should further explore the relationships between rest constructs, fatigue, and physical activity in adults with CFC. Such investigations could provide valuable insights and inform strategies to optimise rest within clinical rehabilitation, ultimately enhancing patient outcomes.

Moreover, although the sample size in the present study exceeded the minimum threshold recommended for questionnaire development and validation using exploratory factor analysis, replication of these findings in larger and more diverse clinical samples is advised. Expanding the research to include additional CFC, such as and traumatic brain injury, would enhance the generalisability of the results and provide further validation of the questionnaire’s psychometric properties, ensuring its applicability across different clinical populations.

## Conclusion

This study aimed to develop and validate the MRES for adults with CFC, utilising a three-phased methodology. The initial Delphi phase produced 25 consensus items, which were subsequently refined through exploratory factor analysis, resulting in a final set of 22 items and a six-factor solution. The findings demonstrate that the MRES-22 is both a valid and reliable tool for assessing rest-related constructs in adults with CFC. More importantly, the findings highlight a framework for evaluating rest-related behaviours, providing valuable insights into resting activity, restorative rest, and rest planning, physical and psychological barriers, and emotional responses to rest. This underscores the importance of adopting a tailored multidimensional approach to effectively managing fatigue. Future research is recommended to explore the relationships between various rest constructs, such as those captured by the MRES-22, and key health outcomes to guide intervention efforts (e.g., activity pacing) for adults with CFC in rehabilitation.

## Supporting information

S1 File PRISMA flowchart for study screening. (DOCX)

S2 File Characteristics of adults with chronic fatiguing conditions and health care professionals involved in the qualitative and Delphi studies. (DOCX)

S3 File Summary results of discriminant validity. (DOCX)
