# Supplementary material for: Development and validation of the rest experience scale for adults with chronically fatiguing conditions
Source: PLoS One. 2026 Sep 2;21(9):e0355730. doi: 10.1371/journal.pone.0355730 (PMC13537572; doi:10.1371/journal.pone.0355730)
Supplement: Supplementary file 1 [file pone.0355730.s001.docx]

Article from the databases (PubMed, CINAHL, and Psych articles): 9393

Duplicate removed:356

Title and abstract screening: 9037

Record excluded: 8941

Studies assessed for eligibility:96

Report excluded: 75

Resting-state EEG studies: 41

Not in HCPs or LTCs: 13

Bed rest: 18

Rest measure in sports: 1

Before 1990: 2

Studies included: 21

**Supplementary file 1:** PRISMA flowchart for study screening
