# Supplementary material for: Development and validation of the rest experience scale for adults with chronically fatiguing conditions
Source: PLoS One. 2026 Sep 2;21(9):e0355730. doi: 10.1371/journal.pone.0355730 (PMC13537572; doi:10.1371/journal.pone.0355730)
Supplement: Supplementary file 2 [file pone.0355730.s002.docx]

**Supplementary 1a:** Characteristics of adults with chronic fatiguing conditions involved in the qualitative and Delphi studies

| **Participants ID** | **Age/years** | **Primary long-term condition** | **FSS** | **Duration**  **of fatigue/yrs.** |
| --- | --- | --- | --- | --- |
| P1 | 30-40 | Long Covid and post-orthostatic tachycardia | 7 | 2 |
| P2 | 40-50 | Long Covid | 6.4 | 3 |
| P3 | 50-60 | Long Covid | 5.7 | 1 |
| P4 | 40-50 | Long Covid | 6.2 | 3 |
| P5 | 50-60 | Long Covid | 6 | 3 |
| P6 | 50-60 | Long Covid | 7 | 3 |
| P7 | 30-40 | Long Covid | 6 | 2 |
| P8 | 30-40 | Long Covid | 6.7 | 3 |
| P9 | 40-50 | Chronic Fatigue Syndrome | 6.6 | 13 |
| P10 | 40-50 | Chronic Fatigue Syndrome | 5 | 3 |
| P11 | 50-60 | Long Covid | 6 | 3 |
| P12 | 50-60 | Arthritis and chronic renal conditions | 7 | 3 |
| P13 | 50-60 | Sjogren's Syndrome | 7 | 3 |
| P14 | 40-50 | Multiple Sclerosis | 7 | 3 |
| P15 | 30-40 | Sjogren's Syndrome | 6 | 3 |
| P16 | 60-70 | Hip and knee osteoarthritis | 6.5 | 2 |
| P17 | 30-40 | Multiple sclerosis | 5.6 | 2 |
| P18 | 50-60 | Osteoarthritis | 4 | 2 |
| P19 | 50-60 | Chronic Fatigue Syndrome | 7 | 2 |
| P20 | 50-60 | Rheumatoid arthritis/chronic fatigue | 7 | 2 |
| P21 | 50-60 | Arthritis/Fibromyalgia | 8 | 30 |
| P22 | 50-60 | Multiple sclerosis | 4 | 23 |
| P23 | 60-70 | Sjogren's Syndrome | 8 | 5 |

FSS=Fatigue Severity Scale

**Supplementary 1b:** Characteristics of Health Care Professionals Involved in the Qualitative and Delphi Studies

| Participants | Gender | Experience at the time of interview | Occupation at the time of interview |
| --- | --- | --- | --- |
| HCP1 | Female | 28 years | Occupational therapist |
| HCP2 | Female | 20 years | Occupational therapist |
| HCP3 | Female | 30 years | Physiotherapist |
| HCP4 | Female | 14 years | Health psychologist |

HCP= Health Care Professional
