# Supplementary material for: Development and validation of the rest experience scale for adults with chronically fatiguing conditions
Source: PLoS One. 2026 Sep 2;21(9):e0355730. doi: 10.1371/journal.pone.0355730 (PMC13537572; doi:10.1371/journal.pone.0355730)
Supplement: Supplementary file 3 [file pone.0355730.s003.docx]

**Supplementary file 2**: Summary results of discriminant validity

| **Dimension** | **ER** | **LOS** | **PP** | **PPB** | **R** | **RR** |
| --- | --- | --- | --- | --- | --- | --- |
| **ER** | - |  |  |  |  |  |
| **LOS** | 0.466 | - |  |  |  |  |
| **PP** | 0.47 | 0.515 | - |  |  |  |
| **PPB** | 0.344 | 0.542 | 0.364 | - |  |  |
| **R** | 0.224 | 0.232 | 0.425 | 0.236 | - |  |
| **RR** | 0.147 | 0.098 | 0.161 | 0.18 | 0.152 | - |

ER=Emotional response to rest, RUR=risk of under-resting, PP=planning and prioritising rest, PPB=Physical and psychological barriers, R=resting, RR=restorative rest
