# Supplementary material for: Development and validation of the rest experience scale for adults with chronically fatiguing conditions
Source: PLoS One. 2026 Sep 2;21(9):e0355730. doi: 10.1371/journal.pone.0355730 (PMC13537572; doi:10.1371/journal.pone.0355730)
Supplement: Supplementary file 4 [file pone.0355730.s004.docx]

In the name of God

Hello dear authors,

I say my congratulation on creating this scale, I read it and suggest some comments to develop your nice work:

- In article title, if you remove the word of multidimension, your title better understand.
- Again, I suggest replace Adults **with** **Long-Term Conditions and Fatigue** with adults with chronically fatigue conditions
- In line 119-120, you wrote, at present, there are relatively few validated scales available to holistically measure the concept, locate it in last paragraph of introduction.
- In method you mentioned **Literature review strategy,** but did not provide Prisma chart, please add it.
- Again, The literature review is described only briefly. Important methodological details are missing: search strategy (databases, date range), inclusion/exclusion criteria, how grey literature was searched, and how data from the review were extracted and synthesized to produce items.
- The threshold for consensus is reported as <70% for non-consensus, but the exact consensus criterion (e.g., ≥70% agreement) should be stated explicitly and justified with references or rationale.
- how “agreement” was operationalised (e.g., combining agree/strongly agree) is not stated.
- Procedures for handling neutral responses and for analyzing open-text comments are not described in sufficient detail.
-  The sample size justification uses the 5:1 rule; however, no actual sample size achieved or recruitment/response rates are provided.
-  No description of sampling frame, recruitment channels, inclusion/exclusion criteria in the survey phase, or participant characteristics (age, sex, LTC type, fatigue severity). This is essential to judge generalisability and to interpret psychometric analyses.
- Provide a table summarising the qualitative interview sample and Delphi panel composition (role, number, country/region if relevant, years of experience, recruitment source).
- Your discussion was good.
