# Supplementary material for: Development and validation of the rest experience scale for adults with chronically fatiguing conditions
Source: PLoS One. 2026 Sep 2;21(9):e0355730. doi: 10.1371/journal.pone.0355730 (PMC13537572; doi:10.1371/journal.pone.0355730)
Supplement: Supplementary file 5 [file pone.0355730.s007.docx]

**Dear editor and reviewer,**

Thank you for the opportunity to resubmit a revised draft of our manuscript, “Development and Validation of the Rest Experience Scale for Adults with Chronically Fatiguing Conditions,” for consideration in PLOS ONE. We are grateful for the time and effort you and the reviewers have invested in providing feedback. The thoughtful comments have been invaluable in enhancing the clarity, rigor, and overall quality of our paper.

We have carefully addressed each reviewer’s comment and made the necessary revisions. On behalf of my co-authors, I am submitting our detailed responses to the reviewers’ insightful comments. In the marked manuscript, all revisions are highlighted in red, and corresponding line numbers are provided for clarity.

Regarding the financial disclosure:

“First author Martin Ackah was funded through a PhD studentship from Northumbria University. The funders had no role in study design, data collection and analysis, decision to publish, or preparation of the manuscript.”

Regarding funding number:

It is important to highlight that this was a PhD studentship funded by Northumbria University, and there was no grant number.

We trust that the revised manuscript reflects these improvements and hope it now meets the journal’s standards.

**Response to reviewers’ comments**

**Comment 1**: Insufficient description of the literature review process. The manuscript states that the literature review methodology has been published elsewhere. However, since the literature review directly informed item generation, a minimal description should be included in the current manuscript. Please briefly describe: databases searched, search timeframe, inclusion/exclusion criteria, and consider including a PRISMA flow diagram (either in the main text or supplementary material).

**Authors’ response**: We thank the reviewer for the comment. We now have revised the manuscript to include a concise description of the literature review process. Specifically, we now outline the databases searched, the search timeframe, and the key inclusion and exclusion criteria. In addition, a PRISMA flow diagram illustrating the study selection process has been included as Supplementary File 1. Please see line 200-211

**Comment** 2. Sample characteristics and generalizability

The study sample shows notable imbalances (e.g., high proportion of females and predominantly White participants), and recruitment was conducted through patient support groups.
Suggestions: Explicitly discuss potential sampling bias in the Discussion section. Comment on the implications for generalizability to broader populations with chronically fatiguing conditions.

**Authors’ response:** We thank the reviewer for the comment. We have expanded the Discussion section to more explicitly address potential sampling bias, including the overrepresentation of female and White participants, as well as recruitment through patient support groups. We have also discussed the implications of these factors for the generalisability of our findings to the broader population of adults with chronically fatiguing conditions. please see line 607-630.

3. Convergent and discriminant validity reporting. The manuscript reports HTMT values and AVE; however, interpretation could be strengthened.

Suggestions: Clarify the threshold used for HTMT (e.g., 0.85 or 0.90) and provide references.
Expand the discussion of validity results to improve interpretability for readers.

**Authors’ response:** We thank the reviewer for the comment. We have now clarified the threshold applied for the HTMT criterion, specifying that values below 0.85 were considered indicative of adequate discriminant validity, and have included the appropriate supporting reference. This amendment has been incorporated into the methods section (line 309). In addition, we have expanded the discussion of the validity findings to improve clarity and interpretability for readers. Specifically, we now provide a more detailed explanation of the AVE and HTMT results and discuss their implications for the multidimensional structure and practical application of MRES-22. These revisions can be found in lines 532–567.

Minor Comments

1. Language and grammar

There are minor grammatical issues throughout the manuscript (e.g., “multidimensional aspect of rest experience” should be revised). Careful proofreading is recommended.

Authors’ response: revised

comment 2. Terminology consistency. Ensure consistent use of terms such as “rest,” “rest experience,” and “restorative rest.”

**Authors’ response:** Thank you for the comment. We appreciate the importance of clear and consistent terminology. In this manuscript, we have used the terms contextually to reflect their specific meaning within different measures and constructs. Specifically, “rest” is used in its general sense and when referring to the established CPCI rest subscale. “Rest experience” refers to the construct as operationalised within the newly developed MRES-22, where it captures participants’ subjective experiences of rest. “Restorative rest” is used to denote a specific component within the MRES-22 subscale, referring to rest that is perceived as physically or mentally restorative. We have reviewed the manuscript to ensure that the use of these terms is clear and consistently applied within their respective contexts, and we believe this contextual distinction is appropriate and justified given the conceptual differences between the constructs.

3. Tables and figures

comment: Ensure all figures (e.g., Fig 1 and Fig 2) are clearly presented and appropriately referenced in the text.

**Authors’ response**: We have carefully reviewed all figures to ensure they are clearly presented, appropriately labelled, and consistently referenced within the text. In particular, we have checked that Figures 1 and 2 are correctly cited at the relevant points in the manuscript and that their presentation enhances clarity and alignment with the corresponding results sections

Comment: Consider improving table readability (e.g., alignment, formatting consistency).

**Authors’ response**: We have reviewed all tables and made revisions to improve readability, including adjustments to alignment, spacing, and overall formatting consistency.

4. Formatting issues

comment: Check citation formatting and spacing inconsistencies and ensure all abbreviations are defined at first use.

**Authors’ response**: Citation and formatting and spacing inconsistencies have been corrected, and all abbreviations are now defined at first mention throughout the manuscript.
