# Supplementary material for: Development and validation of the rest experience scale for adults with chronically fatiguing conditions
Source: PLoS One. 2026 Sep 2;21(9):e0355730. doi: 10.1371/journal.pone.0355730 (PMC13537572; doi:10.1371/journal.pone.0355730)
Supplement: Peer review history

# Peer review: Decision Letter 0

**DOI:** 10.1371/journal.pone.0355730.r001

**Authors:** Jen Edwards

10 Dec 2025

Dear Dr. Deary,

Thank you for submitting your manuscript to PLOS ONE. After careful consideration, we feel that it has merit but does not fully meet PLOS ONE’s publication criteria as it currently stands. Therefore, we invite you to submit a revised version of the manuscript that addresses the points raised during the review process.

The manuscript has been evaluated by one reviewer, and their comments are available below. They have raised concerns with the detail provided in the methodology section and requested further summary of the interview samples.

Could you please revise the manuscript to carefully address the concerns raised?

Please note that we have only been able to secure a single reviewer to assess your manuscript. We are issuing a decision on your manuscript at this point to prevent further delays in the evaluation of your manuscript. Please be aware that the editor who handles your revised manuscript might find it necessary to invite additional reviewers to assess this work once the revised manuscript is submitted. However, we will aim to proceed on the basis of this single review if possible.

Please submit your revised manuscript by Jan 23 2026 11:59PM. If you will need more time than this to complete your revisions, please reply to this message or contact the journal office at plosone@plos.org. When you're ready to submit your revision, log on to https://www.editorialmanager.com/pone/ and select the 'Submissions Needing Revision' folder to locate your manuscript file.

- A rebuttal letter that responds to each point raised by the academic editor and reviewer(s). You should upload this letter as a separate file labeled 'Response to Reviewers'.
- A marked-up copy of your manuscript that highlights changes made to the original version. You should upload this as a separate file labeled 'Revised Manuscript with Track Changes'.
- An unmarked version of your revised paper without tracked changes. You should upload this as a separate file labeled 'Manuscript'.

If you would like to make changes to your financial disclosure, please include your updated statement in your cover letter. Guidelines for resubmitting your figure files are available below the reviewer comments at the end of this letter.

If applicable, we recommend that you deposit your laboratory protocols in protocols.io to enhance the reproducibility of your results. Protocols.io assigns your protocol its own identifier (DOI) so that it can be cited independently in the future. For instructions see: https://journals.plos.org/plosone/s/submission-guidelines#loc-laboratory-protocols. Additionally, PLOS ONE offers an option for publishing peer-reviewed Lab Protocol articles, which describe protocols hosted on protocols.io. Read more information on sharing protocols at https://plos.org/protocols?utm_medium=editorial-email&utm_source=authorletters&utm_campaign=protocols.

We look forward to receiving your revised manuscript.

Kind regards,

Jen Edwards

Staff Editor

PLOS One

Journal Requirements:

When submitting your revision, we need you to address these additional requirements.

1. Please ensure that your manuscript meets PLOS ONE's style requirements, including those for file naming. The PLOS ONE style templates can be found at

https://journals.plos.org/plosone/s/file?id=wjVg/PLOSOne_formatting_sample_main_body.pdf and

https://journals.plos.org/plosone/s/file?id=ba62/PLOSOne_formatting_sample_title_authors_affiliations.pdf

2. Thank you for stating the following financial disclosure:

“First author Martin Ackah was funded through a PhD studentship from Northumbria University.”

Please state what role the funders took in the study. If the funders had no role, please state: "The funders had no role in study design, data collection and analysis, decision to publish, or preparation of the manuscript."

If this statement is not correct you must amend it as needed.

Please include this amended Role of Funder statement in your cover letter; we will change the online submission form on your behalf.

3. If the reviewer comments include a recommendation to cite specific previously published works, please review and evaluate these publications to determine whether they are relevant and should be cited. There is no requirement to cite these works unless the editor has indicated otherwise.

[Note: HTML markup is below. Please do not edit.]

Reviewers' comments:

Reviewer's Responses to Questions

**Comments to the Author**

1. Is the manuscript technically sound, and do the data support the conclusions?

Reviewer #1: Yes

**********

2. Has the statistical analysis been performed appropriately and rigorously?

Reviewer #1: Yes

**********

3. Have the authors made all data underlying the findings in their manuscript fully available?

The PLOS Data policy

Reviewer #1: Yes

**********

4. Is the manuscript presented in an intelligible fashion and written in standard English?

Reviewer #1: Yes

**********

Reviewer #1: In the name of God

Hello dear authors,

I say my congratulation on creating this scale, I read it and suggest some comments to develop your nice work:

• In article title, if you remove the word of multidimension, your title better understand.

• Again, I suggest replace Adults with Long-Term Conditions and Fatigue with adults with chronically fatigue conditions

• In line 119-120, you wrote, at present, there are relatively few validated scales available to holistically measure the concept, locate it in last paragraph of introduction.

• In method you mentioned Literature review strategy, but did not provide Prisma chart, please add it.

• Again, The literature review is described only briefly. Important methodological details are missing: search strategy (databases, date range), inclusion/exclusion criteria, how grey literature was searched, and how data from the review were extracted and synthesized to produce items.

• The threshold for consensus is reported as <70% for non-consensus, but the exact consensus criterion (e.g., ≥70% agreement) should be stated explicitly and justified with references or rationale.

• how “agreement” was operationalised (e.g., combining agree/strongly agree) is not stated.

• Procedures for handling neutral responses and for analyzing open-text comments are not described in sufficient detail.

• • The sample size justification uses the 5:1 rule; however, no actual sample size achieved or recruitment/response rates are provided.

• • No description of sampling frame, recruitment channels, inclusion/exclusion criteria in the survey phase, or participant characteristics (age, sex, LTC type, fatigue severity). This is essential to judge generalisability and to interpret psychometric analyses.

• Provide a table summarising the qualitative interview sample and Delphi panel composition (role, number, country/region if relevant, years of experience, recruitment source).

• Your discussion was good.

**********

what does this mean?). If published, this will include your full peer review and any attached files.

If you choose “no”, your identity will remain anonymous but your review may still be made public.

**Do you want your identity to be public for this peer review?**  For information about this choice, including consent withdrawal, please see our Privacy Policy

Reviewer #1: **Yes:** dr mitra khoobi assistant professor of nursing education.

**********

[NOTE: If reviewer comments were submitted as an attachment file, they will be attached to this email and accessible via the submission site. Please log into your account, locate the manuscript record, and check for the action link "View Attachments". If this link does not appear, there are no attachment files.]

To ensure your figures meet our technical requirements, please review our figure guidelines: https://journals.plos.org/plosone/s/figures

You may also use PLOS’s free figure tool, NAAS, to help you prepare publication quality figures: https://journals.plos.org/plosone/s/figures#loc-tools-for-figure-preparation.

NAAS will assess whether your figures meet our technical requirements by comparing each figure against our figure specifications.

Attachment Submitted filename: plose one.1azar.docx

# Author comment: Author response to Decision Letter 1

**DOI:** 10.1371/journal.pone.0355730.r002

15 Jan 2026

Response to reviewer’s comments

Reviewer #1:

Comment 1: In article title, if you remove the word multidimension, your title better understand.

Authors’ response: We thank the reviewer for this suggestion. The term “multidimension” has been removed, and the title now reads: “Development and Validation of the Rest Experience Scale for Adults with Chronically Fatiguing Conditions.” Please see line 1-2

comment 2: Again, I suggest replacing Adults with Long-Term Conditions and Fatigue with adults with chronically fatigued conditions.

Authors’ response: We agree with the reviewer and have accordingly replaced “adults with long-term conditions with fatigue” with “adults with chronically fatiguing conditions.” This change has been reflected throughout the manuscript.

comment: In method you mentioned Literature review strategy, but did not provide Prisma chart, please add it.

Authors’ response: Thank you for the comment. Please see the next/below authors’ response.

comment: Again, The literature review is described only briefly. Important methodological details are missing: search strategy (databases, date range), inclusion/exclusion criteria, and how data from the review were extracted and synthesized to produce items.

Authors’ response: We thank the reviewer for this constructive comment. The literature review phase, including the full search strategy, inclusion and exclusion criteria, procedures, and data synthesis methods, has been comprehensively reported in a previously published manuscript (Ackah et al., 2025). To ensure clarity and transparency for readers, we have now revised the corresponding sentence in the manuscript and added the appropriate citation to reference the detailed methodology. Please see line 196-198

comment: The threshold for consensus is reported as <70% for non-consensus, but the exact consensus criterion (e.g., ≥70% agreement) should be stated explicitly and justified with references or rationale.

Authors’ response: Thank you for this comment. Consensus was defined as a priori as agreement by ≥70% of participants. Therefore, items with <70% agreement were classified as non-consensus (Please see line 217). This criterion was applied consistently and is reported in the Phase one analysis (Please see line 270-274). We have now clarified this explicitly in the manuscript to improve transparency

comment: how “agreement” was operationalised (e.g., combining agree/strongly agree) is not stated.

Authors’ response: We thank the reviewer for this comment. Agreement was operationalised by combining responses of “agree” and “strongly agree”, as described in the Phase one analysis To improve clarity, we have refined the relevant sentence in the manuscript to state this explicitly (Please see line 270-274).

comment: Procedures for handling neutral responses and for analyzing open-text comments are not described in sufficient detail.

Authors’ response: We thank the reviewer for this comment. We have now provided additional detail on the handling of neutral responses (Please see line 270-275)) and the analysis of open-text comments (Please see line 218-222).

comment: The sample size justification uses the 5:1 rule; however, no actual sample size achieved, or recruitment/response rates are provided.

Authors’ response: We thank the reviewer for this comment. The sample size was determined using the guideline of five participants per item (Anthoine et al., 2014; Costello & Osborne, 2005; Sook et al., 2018). The questionnaire employed in this study contains 25 items; therefore, a minimum of 125 participants (5 × 25) were required (please line 239-244). In this phase, a total of 162 participants were recruited, exceeding the minimum requirement and ensuring sufficient power for the psychometric analyses.

comment: No description of sampling frame, recruitment channels, inclusion/exclusion criteria in the survey phase, or participant characteristics (age, sex, LTC type, fatigue severity). This is essential to judge generalisability and to interpret psychometric analyses.

Authors’ response: We thank the reviewer for this comment. Participants in the survey phase were previously noted to be required to meet the same eligibility criteria for adults with chronic conditions and fatigue as outlined in the modified Delphi study, to avoid repetition. Nevertheless, we have now provided a more detailed description of the sampling frame, recruitment channels, inclusion and exclusion criteria, and participant characteristics (please see lines 233–238).

comment: Provide a table summarising the qualitative interview sample and Delphi panel composition (role, number, country/region if relevant, years of experience, recruitment source).

Authors’ response: We thank the reviewer for this comment. We have now provided a table summarising the qualitative interview sample and Delphi panel composition in Supplementary File 1 (Please see lines 202, 313, and 901-902).

comment: Your discussion was good.

Authors’ response: Thank you

# Peer review: Decision Letter 1

**DOI:** 10.1371/journal.pone.0355730.r003

**Authors:** Lanlan Chen

27 Apr 2026

Dear Dr. Deary,

Thank you for submitting your manuscript to PLOS ONE. After careful consideration, we feel that it has merit but does not fully meet PLOS ONE’s publication criteria as it currently stands. Therefore, we invite you to submit a revised version of the manuscript that addresses the points raised during the review process.

Please submit your revised manuscript by Jun 11 2026 11:59PM. If you will need more time than this to complete your revisions, please reply to this message or contact the journal office at plosone@plos.org. When you're ready to submit your revision, log on to https://www.editorialmanager.com/pone/ and select the 'Submissions Needing Revision' folder to locate your manuscript file.

- A letter that responds to each point raised by the academic editor and reviewer(s). You should upload this letter as a separate file labeled 'Response to Reviewers'.
- A marked-up copy of your manuscript that highlights changes made to the original version. You should upload this as a separate file labeled 'Revised Manuscript with Track Changes'.
- An unmarked version of your revised paper without tracked changes. You should upload this as a separate file labeled 'Manuscript'.

If you would like to make changes to your financial disclosure, please include your updated statement in your cover letter. Guidelines for resubmitting your figure files are available below the reviewer comments at the end of this letter.

If applicable, we recommend that you deposit your laboratory protocols in protocols.io to enhance the reproducibility of your results. Protocols.io assigns your protocol its own identifier (DOI) so that it can be cited independently in the future. For instructions see: https://journals.plos.org/plosone/s/submission-guidelines#loc-laboratory-protocols. Additionally, PLOS ONE offers an option for publishing peer-reviewed Lab Protocol articles, which describe protocols hosted on protocols.io. Read more information on sharing protocols at https://plos.org/protocols?utm_medium=editorial-email&utm_source=authorletters&utm_campaign=protocols.

As the corresponding author, your ORCID iD is verified in the submission system and will appear in the published article. PLOS supports the use of ORCID, and we encourage all coauthors to register for an ORCID iD and use it as well. Please encourage your coauthors to verify their ORCID iD within the submission system before final acceptance, as unverified ORCID iDs will not appear in the published article. *Only*  the individual author can complete the verification step; PLOS staff *cannot*  verify ORCID iDs on behalf of authors.

We look forward to receiving your revised manuscript.

Kind regards,

Lanlan Chen

Academic Editor

PLOS One

Journal Requirements:

If the reviewer comments include a recommendation to cite specific previously published works, please review and evaluate these publications to determine whether they are relevant and should be cited. There is no requirement to cite these works unless the editor has indicated otherwise.

Please review your reference list to ensure that it is complete and correct. If you have cited papers that have been retracted, please include the rationale for doing so in the manuscript text, or remove these references and replace them with relevant current references. Any changes to the reference list should be mentioned in the rebuttal letter that accompanies your revised manuscript. If you need to cite a retracted article, indicate the article’s retracted status in the References list and also include a citation and full reference for the retraction notice.

[Note: HTML markup is below. Please do not edit.]

Reviewers' comments:

Reviewer's Responses to Questions

**Comments to the Author**

Reviewer #2: (No Response)

**********

2. Is the manuscript technically sound, and do the data support the conclusions?

Reviewer #2: Yes

**********

3. Has the statistical analysis been performed appropriately and rigorously?

Reviewer #2: Yes

**********

4. Have the authors made all data underlying the findings in their manuscript fully available?

The PLOS Data policy

Reviewer #2: Yes

**********

5. Is the manuscript presented in an intelligible fashion and written in standard English?

Reviewer #2: Yes

**********

Reviewer #2: Reviewer Comments

General Comments

This manuscript presents the development and validation of the Multidimensional Rest Experience Scale (MRES) for adults with chronically fatiguing conditions. The topic is relevant and timely, as “rest” remains an under-conceptualized and under-measured construct in fatigue management and rehabilitation research. The authors followed a structured approach including a modified Delphi process, exploratory factor analysis, and reliability testing. Overall, the study is of potential interest and provides a useful contribution to the field. The methodology is generally sound, and the results demonstrate acceptable psychometric properties. However, several aspects of the manuscript require clarification and minor improvement before it is suitable for publication.

Major Comments

1. Insufficient description of the literature review process

The manuscript states that the literature review methodology has been published elsewhere. However, since the literature review directly informed item generation, a minimal description should be included in the current manuscript. Please briefly describe: databases searched, search timeframe, inclusion/exclusion criteria, and consider including a PRISMA flow diagram (either in the main text or supplementary material)

2. Sample characteristics and generalizability

The study sample shows notable imbalances (e.g., high proportion of females and predominantly White participants), and recruitment was conducted through patient support groups.

Suggestions: Explicitly discuss potential sampling bias in the Discussion section. Comment on the implications for generalizability to broader populations with chronically fatiguing conditions.

3. Convergent and discriminant validity reporting

The manuscript reports HTMT values and AVE; however, interpretation could be strengthened.

Suggestions:

Clarify the threshold used for HTMT (e.g., 0.85 or 0.90) and provide references.

Expand the discussion of validity results to improve interpretability for readers.

Minor Comments

1. Language and grammar

There are minor grammatical issues throughout the manuscript (e.g., “multidimensional aspect of rest experience” should be revised).

Careful proofreading is recommended.

2. Terminology consistency

Ensure consistent use of terms such as “rest,” “rest experience,” and “restorative rest.”

3. Tables and figures

Ensure all figures (e.g., Fig 1 and Fig 2) are clearly presented and appropriately referenced in the text.

Consider improving table readability (e.g., alignment, formatting consistency).

4. Formatting issues

Check citation formatting and spacing inconsistencies.

Ensure all abbreviations are defined at first use.

**********

what does this mean?). If published, this will include your full peer review and any attached files.

If you choose “no”, your identity will remain anonymous but your review may still be made public.

**Do you want your identity to be public for this peer review?**  For information about this choice, including consent withdrawal, please see our Privacy Policy

Reviewer #2: No

**********

[NOTE: If reviewer comments were submitted as an attachment file, they will be attached to this email and accessible via the submission site. Please log into your account, locate the manuscript record, and check for the action link "View Attachments". If this link does not appear, there are no attachment files.]

To ensure your figures meet our technical requirements, please review our figure guidelines: https://journals.plos.org/plosone/s/figures

You may also use PLOS’s free figure tool, NAAS, to help you prepare publication quality figures: https://journals.plos.org/plosone/s/figures#loc-tools-for-figure-preparation.

NAAS will assess whether your figures meet our technical requirements by comparing each figure against our figure specifications.

# Author comment: Author response to Decision Letter 2

**DOI:** 10.1371/journal.pone.0355730.r004

21 Jul 2026

Dear editor and reviewers,

Thank you for the opportunity to resubmit a revised draft of our manuscript, “‘’ Development and Validation of the Rest Experience Scale for Adults with Chronically Fatiguing Conditions” for publication in PLOS ONE. We are grateful for the time and effort you and the reviewers have invested in providing feedback. We truly appreciate the thoughtful comments, which have been invaluable in enhancing our paper. We have carefully addressed each of the reviewers' concerns and made the necessary revisions. On behalf of my co-authors, I am submitting our detailed response to the reviewers' insightful comments. Below, we have outlined our responses to each comment, with corresponding line numbers provided in the marked manuscript. All revisions are highlighted in red. We trust that the revised manuscript has improved through this process and hope it now meets the journal’s standards.

Response to reviewers’ comments

Comment 1: Insufficient description of the literature review process. The manuscript states that the literature review methodology has been published elsewhere. However, since the literature review directly informed item generation, a minimal description should be included in the current manuscript. Please briefly describe: databases searched, search timeframe, inclusion/exclusion criteria, and consider including a PRISMA flow diagram (either in the main text or supplementary material).

Authors’ response: We thank the reviewer for the comment. We now have revised the manuscript to include a concise description of the literature review process. Specifically, we now outline the databases searched, the search timeframe, and the key inclusion and exclusion criteria. In addition, a PRISMA flow diagram illustrating the study selection process has been included as Supplementary File 1. Please see line 200-211

Comment 2. Sample characteristics and generalizability

The study sample shows notable imbalances (e.g., high proportion of females and predominantly White participants), and recruitment was conducted through patient support groups.

Suggestions: Explicitly discuss potential sampling bias in the Discussion section. Comment on the implications for generalizability to broader populations with chronically fatiguing conditions.

Authors’ response: We thank the reviewer for the comment. We have expanded the Discussion section to more explicitly address potential sampling bias, including the overrepresentation of female and White participants, as well as recruitment through patient support groups. We have also discussed the implications of these factors for the generalisability of our findings to the broader population of adults with chronically fatiguing conditions. please see line 607-630.

3. Convergent and discriminant validity reporting. The manuscript reports HTMT values and AVE; however, interpretation could be strengthened.

Suggestions: Clarify the threshold used for HTMT (e.g., 0.85 or 0.90) and provide references.

Expand the discussion of validity results to improve interpretability for readers.

Authors’ response: We thank the reviewer for the comment. We have now clarified the threshold applied for the HTMT criterion, specifying that values below 0.85 were considered indicative of adequate discriminant validity, and have included the appropriate supporting reference. This amendment has been incorporated into the methods section (line 309). In addition, we have expanded the discussion of the validity findings to improve clarity and interpretability for readers. Specifically, we now provide a more detailed explanation of the AVE and HTMT results and discuss their implications for the multidimensional structure and practical application of MRES-22. These revisions can be found in lines 532–567.

Minor Comments

1. Language and grammar

There are minor grammatical issues throughout the manuscript (e.g., “multidimensional aspect of rest experience” should be revised). Careful proofreading is recommended.

Authors’ response: revised

comment 2. Terminology consistency. Ensure consistent use of terms such as “rest,” “rest experience,” and “restorative rest.”

Authors’ response: Thank you for the comment. We appreciate the importance of clear and consistent terminology. In this manuscript, we have used the terms contextually to reflect their specific meaning within different measures and constructs. Specifically, “rest” is used in its general sense and when referring to the established CPCI rest subscale. “Rest experience” refers to the construct as operationalised within the newly developed MRES-22, where it captures participants’ subjective experiences of rest. “Restorative rest” is used to denote a specific component within the MRES-22 subscale, referring to rest that is perceived as physically or mentally restorative.

Response: We have reviewed the manuscript to ensure that the use of these terms is clear and consistently applied within their respective contexts, and we believe this contextual distinction is appropriate and justified given the conceptual differences between the constructs.

3. Tables and figures

comment: Ensure all figures (e.g., Fig 1 and Fig 2) are clearly presented and appropriately referenced in the text.

Authors’ response: We have carefully reviewed all figures to ensure they are clearly presented, appropriately labelled, and consistently referenced within the text. In particular, we have checked that Figures 1 and 2 are correctly cited at the relevant points in the manuscript and that their presentation enhances clarity and alignment with the corresponding results sections

Comment: Consider improving table readability (e.g., alignment, formatting consistency).

Authors’ response: We have reviewed all tables and made revisions to improve readability, including adjustments to alignment, spacing, and overall formatting consistency.

4. Formatting issues

comment: Check citation formatting and spacing inconsistencies and ensure all abbreviations are defined at first use.

Authors’ response: Citation and formatting and spacing inconsistencies have been corrected, and all abbreviations are now defined at first mention throughout the manuscript.

First author Martin Ackah was funded through a PhD studentship from Northumbria University. (NB: there was no grant number associated with this.) The funders had no role in study design, data collection and analysis, decision to publish, or preparation of the manuscript.

Attachment Submitted filename: Responses to reviewers comment2107.docx

# Peer review: Decision Letter 2

**DOI:** 10.1371/journal.pone.0355730.r005

**Authors:** Lanlan Chen

26 Jul 2026

Development and Validation of the Rest Experience Scale for Adults with Chronically Fatiguing Conditions

PONE-D-25-51926R2

Dear Dr. Deary,

We’re pleased to inform you that your manuscript has been judged scientifically suitable for publication and will be formally accepted for publication once it meets all outstanding technical requirements.

Within one week, you’ll receive an e-mail detailing the required amendments. When these have been addressed, you’ll receive a formal acceptance letter and your manuscript will be scheduled for publication.

An invoice will be generated when your article is formally accepted. Please note, if your institution has a publishing partnership with PLOS and your article meets the relevant criteria, all or part of your publication costs will be covered. Please make sure your user information is up-to-date by logging into Editorial Manager at Editorial Manager® and clicking the ‘Update My Information' link at the top of the page. For questions related to billing, please contact billing support.

If your institution or institutions have a press office, please notify them about your upcoming paper to help maximize its impact. If they’ll be preparing press materials, please inform our press team as soon as possible -- no later than 48 hours after receiving the formal acceptance. Your manuscript will remain under strict press embargo until 2 pm Eastern Time on the date of publication. For more information, please contact onepress@plos.org.

Kind regards,

Lanlan Chen

Academic Editor

PLOS One

Additional Editor Comments (optional):

Reviewers' comments:

Reviewer's Responses to Questions

**Comments to the Author**

Reviewer #2: All comments have been addressed

**********

2. Is the manuscript technically sound, and do the data support the conclusions?

Reviewer #2: Yes

**********

3. Has the statistical analysis been performed appropriately and rigorously?

Reviewer #2: Yes

**********

4. Have the authors made all data underlying the findings in their manuscript fully available?

The PLOS Data policy

Reviewer #2: No

**********

5. Is the manuscript presented in an intelligible fashion and written in standard English?

Reviewer #2: Yes

**********

Reviewer #2: The authors have carefully addressed all the comments and concerns raised in my previous review. The revised manuscript has been substantially improved in terms of clarity, methodological explanation, presentation of the results, and overall organization. The authors have provided detailed and satisfactory responses to each of my comments, and the corresponding revisions have been appropriately incorporated into the manuscript. In my opinion, the major issues identified during the initial review have now been adequately resolved, and no further substantial revisions are required. The manuscript is scientifically sound, clearly written, and suitable for publication in its current form. Therefore, I recommend that the manuscript be accepted for publication.

**********

what does this mean?). If published, this will include your full peer review and any attached files.

If you choose “no”, your identity will remain anonymous but your review may still be made public.

**Do you want your identity to be public for this peer review?**  For information about this choice, including consent withdrawal, please see our Privacy Policy

Reviewer #2: No

**********

# Editor report: Acceptance letter

**DOI:** 10.1371/journal.pone.0355730.r006

**Authors:** Lanlan Chen

PONE-D-25-51926R2

PLOS One

Dear Dr. Deary,

I'm pleased to inform you that your manuscript has been deemed suitable for publication in PLOS One. Congratulations! Your manuscript is now being handed over to our production team.

At this stage, our production department will prepare your paper for publication. This includes ensuring the following:

* All references, tables, and figures are properly cited

* All relevant supporting information is included in the manuscript submission,

* There are no issues that prevent the paper from being properly typeset

You will receive further instructions from the production team, including instructions on how to review your proof when it is ready. Please keep in mind that we are working through a large volume of accepted articles, so please give us a few days to review your paper and let you know the next and final steps.

Lastly, if your institution or institutions have a press office, please let them know about your upcoming paper now to help maximize its impact. If they'll be preparing press materials, please inform our press team within the next 48 hours. Your manuscript will remain under strict press embargo until 2 pm Eastern Time on the date of publication. For more information, please contact onepress@plos.org.

You will receive an invoice from PLOS for your publication fee after your manuscript has reached the completed accept phase. If you receive an email requesting payment before acceptance or for any other service, this may be a phishing scheme. Learn how to identify phishing emails and protect your accounts at https://explore.plos.org/phishing.

If we can help with anything else, please email us at customercare@plos.org.

Thank you for submitting your work to PLOS One and supporting open access.

Kind regards,

PLOS ONE Editorial Office Staff

on behalf of

M.D Lanlan Chen

Academic Editor

PLOS One
